# Serotonergic Modulation of Locomotor Activity From Basal Vertebrates to Mammals

**DOI:** 10.3389/fncir.2020.590299

**Published:** 2020-11-05

**Authors:** Aurélie Flaive, Maxime Fougère, Cornelis Immanuel van der Zouwen, Dimitri Ryczko

**Affiliations:** ^1^Département de Pharmacologie-Physiologie, Faculté de Médecine et des Sciences de la Santé, Université de Sherbrooke, Sherbrooke, QC, Canada; ^2^Centre de Recherche du Centre Hospitalier Universitaire de Sherbrooke, Sherbrooke, QC, Canada; ^3^Institut de Pharmacologie de Sherbrooke, Sherbrooke, QC, Canada; ^4^Centre des Neurosciences de Sherbrooke, Sherbrooke, QC, Canada

**Keywords:** serotonin, locomotion, spinal cord, brainstem, raphe nuclei

## Abstract

During the last 50 years, the serotonergic (5-HT) system was reported to exert a complex modulation of locomotor activity. Here, we focus on two key factors that likely contribute to such complexity. First, locomotion is modulated directly and indirectly by 5-HT neurons. The locomotor circuitry is directly innervated by 5-HT neurons in the caudal brainstem and spinal cord. Also, indirect control of locomotor activity results from ascending projections of 5-HT cells in the rostral brainstem that innervate multiple brain centers involved in motor action planning. Second, each approach used to manipulate the 5-HT system likely engages different 5-HT-dependent mechanisms. This includes the recruitment of different 5-HT receptors, which can have excitatory or inhibitory effects on cell activity. These receptors can be located far or close to the 5-HT release sites, making their activation dependent on the level of 5-HT released. Here we review the activity of different 5-HT nuclei during locomotor activity, and the locomotor effects of 5-HT precursors, exogenous 5-HT, selective 5-HT reuptake inhibitors (SSRI), electrical or chemical stimulation of 5-HT neurons, genetic deletions, optogenetic and chemogenetic manipulations. We highlight both the coherent and controversial aspects of 5-HT modulation of locomotor activity from basal vertebrates to mammals. This mini review may hopefully inspire future studies aiming at dissecting the complex effects of 5-HT on locomotor function.

## Introduction

The role of the serotonergic (5-HT) system is well known to be an enigma (Jacobs and Fornal, 1995). 5-HT modulates animal cognition and behavior, including motor function, in a complex manner (for review Bacqué-Cazenave et al., 2020). Historically, the interest toward the study of 5-HT modulation of locomotor circuits followed the pioneering observations in Paris by Viala and Buser (1969), who showed that systemic injection of the 5-HT precursor 5-hydroxytryptophan (5-HTP) evoked fictive locomotor activity in rabbits (Viala and Buser, 1969, 1971; see “5-HT Precursors” section). Many studies then reported the powerful excitatory effect of exogenous 5-HT on the fictive locomotor activity produced by the spinal central pattern generator (CPG) for locomotion (see “Exogenous 5-HT” section). These studies uncovered the effects of 5-HT at the cellular level in the spinal locomotor circuitry. However, some unexpected results were reported when using other tools. Increasing the release of endogenous 5-HT with selective 5-HT reuptake inhibitors (SSRI) resulted in the destabilization of fictive locomotor activity in several studies (see “Endogenous 5-HT” section). Genetic tools were then used to delete 5-HT neurons, and this resulted, intriguingly, in almost no effect on locomotor activity (see “Genetic Ablations” section). Recently, the activity of genetically identified 5-HT neurons was reported using *in vivo* calcium imaging, and their activity during ongoing locomotion was manipulated with optogenetics and chemogenetics (see “Manipulating Spinal 5-HT Neuron Activity,” “Recording and Manipulating 5-HT Neuron Activity in the Brainstem Caudal Group,” “Recording and Manipulating 5-HT Neuron Activity in the Brainstem Rostral Group” sections). This uncovered a complex balance of excitatory and inhibitory effects on locomotor activity that: (i) display short- and long-term effects; (ii) depend on the 5-HT nucleus targeted; and (iii) depend on the environmental context.

Here, we briefly present the locomotor circuitry and the 5-HT system and provide an overview of the locomotor effects obtained when using pharmacological and genetic manipulations of the 5-HT system. We highlight the controversies and the possible future developments. Other important aspects of 5-HT modulation include its role during spinal network development (for review Jean-Xavier et al., 2018; Hachoumi and Sillar, 2020), the specific contributions of the different 5-HT receptor subtypes and the plasticity of 5-HT modulation after spinal cord injury (for review Sławińska et al., 2014b; Ghosh and Pearse, 2015; Perrier and Cotel, 2015; Perrin and Noristani, 2019; Sławińska and Jordan, 2019). For further reading on the role of 5-HT in spinal cord-transected animal models and complete spinal cord-injured patients, we refer readers to the studies of the laboratories of e.g., Courtine, Edgerton, Guertin, Jordan, Orsal, or Slawinska.

## The Locomotor System

**Figure 1:**
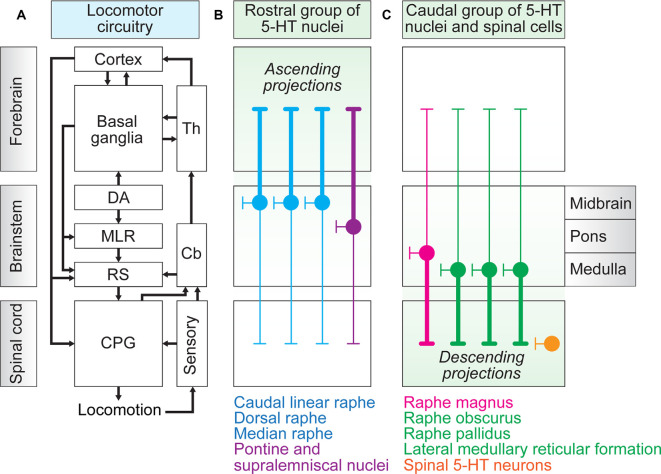
The locomotor circuitry and the serotonergic (5-HT) neurons. **(A)** Schematic representation of the elements constituting the locomotor circuitry, their location in the nervous system, and their connectivity illustrated with black arrows (for review Grillner and El Manira, 2020). **(B)** Schematic representation of the rostral group of 5-HT nuclei (for review Hornung, 2010). These include, in the midbrain, the caudal linear raphe nucleus, the dorsal raphe nucleus, the median raphe nucleus, and in the rostral pons, the pontine and supralemniscal nuclei. The rostral group mostly send ascending projections to the forebrain (thick lines), but also a limited amount of descending projections to the spinal cord (thin lines), and local projections in the brainstem. **(C)** Schematic representation of the caudal group of brainstem 5-HT nuclei and the spinal 5-HT neurons (for review Hornung, 2010). These include the nucleus raphe magnus at the border of the pons and medulla oblongata, and in the medulla oblongata, the nucleus raphe obscurus, nucleus raphe pallidus, and 5-HT neurons of the lateral medullary reticular formation (lateral paragigantocellular nucleus, LPGi). The caudal 5-HT nuclei mainly send descending projections to the spinal cord, but also a limited amount of ascending projections to the forebrain, and local projections to many regions of the brainstem, as well as to the cerebellum. Finally, the spinal 5-HT neurons send local projections rostrally, caudally, and laterally in the spinal cord. Cb, cerebellum, CPG, Central Pattern Generator, DA, dopaminergic neurons, MLR, Mesencephalic Locomotor Region, RS, reticulospinal neurons, TH, thalamus.

The architecture of the locomotor system is largely conserved in vertebrates (Figure 1A). Briefly, the rhythmic and coordinated patterns of muscle activation are generated by a specialized spinal CPG (for review Kiehn, 2016; Grillner and El Manira, 2020). This network integrates sensory feedback, which feeds and shapes the locomotor rhythm and pattern (Böhm et al., 2016; Knafo et al., 2017; for review Frigon, 2017; Knafo and Wyart, 2018). The spinal CPG for locomotion receives descending inputs from brainstem reticulospinal neurons, which constitute the final common descending pathway (Dubuc et al., 2008). They can transmit multiple commands to spinal locomotor circuits, including locomotion initiation commands (Brocard and Dubuc, 2003; Brocard et al., 2010; Hägglund et al., 2010; Bretzner and Brownstone, 2013; Capelli et al., 2017), stop commands (Bouvier et al., 2015; Juvin et al., 2016; Grätsch et al., 2019), and steering and equilibrium control commands (Deliagina et al., 2000; Ryczko et al., 2016c; Cregg et al., 2020). Reticulospinal neurons integrate sensory inputs and carry the locomotor command sent monosynaptically by the Mesencephalic Locomotor Region (MLR), a brainstem region that controls the initiation and speed of locomotion (Sirota et al., 2000; Ryczko et al., 2016a; Caggiano et al., 2018; Josset et al., 2018; for review Ryczko and Dubuc, 2013). The MLR is under tonic inhibitory control of the output structures of the basal ganglia (Roseberry et al., 2016), which are involved in action selection, as well as planning and executing of gait modifications (Stephenson-Jones et al., 2011; Mullié et al., 2020; for review Grillner and Robertson, 2016). Dopaminergic nuclei influence the activity of the MLR indirectly through ascending projections to the basal ganglia (Kravitz et al., 2010), and directly through descending projections to the MLR (Ryczko et al., 2013, 2016b; Pérez-Fernández et al., 2014; Sharma et al., 2018), and the spinal cord (Koblinger et al., 2018; for review Fougère et al., 2019). The motor cortex is involved in precise foot placement by its projections to reticulospinal neurons and spinal CPG neurons (for review Drew and Marigold, 2015). The cerebellum compares the efferent copies of the locomotor commands and sensory information resulting from movement execution and is involved in the learning process underlying the adaptation of the locomotor pattern to environmental perturbations, likely through projections to thalamocortical regions (Darmohray et al., 2019). The cerebellum can also evoke locomotion through projections to the brainstem (Mori et al., 1998; for review Takakusaki, 2017).

## The 5-HT System

5-HT neurons are located in the brainstem and spinal cord and innervate all levels of the locomotor circuitry (Figures 1B,C). In the brainstem, the 5-HT cell bodies are distributed along the midline in the raphe nuclei, which are constituted of two groups. The rostral group is located in the midbrain (caudal linear raphe nucleus, dorsal raphe nucleus, median raphe nucleus) and rostral pons (pontine and supralemniscal nuclei; Jacobs and Azmitia, 1992; Hornung, 2010; Figure 1B). These nuclei mostly send ascending projections to the forebrain, local projections to the brainstem regions, and a limited number of descending projections to the spinal cord (Hornung, 2010). The caudal group is located in the caudal pons (nucleus raphe magnus) and medulla oblongata (nucleus raphe obscurus, nucleus raphe pallidus, and 5-HT neurons of the lateral medullary reticular formation, i.e., lateral paragigantocellularis nucleus, LPGi; Figure 1C). This caudal group mainly sends descending projections to the spinal cord, local projections to the brainstem, and a limited amount of ascending projections to the forebrain (Hornung, 2010). Finally, 5-HT neurons in the spinal cord project locally, send branches rostrally or caudally, or toward the ventrolateral border, and occasionally in the central canal (lamprey: Harris-Warrick et al., 1985; zebrafish: Montgomery et al., 2018; salamander: Flaive et al., 2020; turtle: Fabbiani et al., 2018; mouse: Ballion et al., 2002; Figure 1C). In recent years, the use of genetic tools has allowed researchers to refine the characterization of 5-HT nuclei. In the dorsal raphe nucleus (the largest 5-HT nucleus), subtypes of 5-HT neurons with distinct molecular, electrophysiological, and anatomical signatures have been identified, with e.g., one cell type that selectively innervates the basal ganglia in mice (Ren et al., 2018; Cardozo Pinto et al., 2019; Huang et al., 2019; Okaty et al., 2020).

## 5-HT Modulation of Locomotor Activity

### 5-HT Precursors

Pioneering observations showed that systemic injection of 5-hydroxytryptophan (5-HTP) evokes fictive locomotor activity (i.e., alternation of rhythmic activity in flexor and extensor limb nerves) in anesthetized rabbits (Viala and Buser, 1969), and fictive locomotion in acute spinalized rabbits (Viala and Buser, 1971). 5-HTP does not evoke locomotion in acute spinalized cats (Grillner and Shik, 1973) but increases locomotor burst amplitude during walking in chronic spinalized cats (Barbeau and Rossignol, 1990) and during fictive swimming in frog embryos (Woolston et al., 1994).

### Exogenous 5-HT

The extracellular level of 5-HT can be increased by application of exogenous 5-HT (i.e., a solution of 5-HT prepared by the experimenter) or by blocking the recapture of 5-HT at 5-HT synapses using e.g., SSRI. These two approaches have different effects on the 5-HT system (see “Endogenous 5-HT” section) and therefore, it is important to compare the effects of exogenous and endogenous 5-HT. The powerful excitatory effect of exogenous 5-HT on the spinal locomotor circuitry is well known. Its canonical effect is an increase of locomotor burst intensity and rhythm stability, and a slow down of the fictive locomotor rhythm evoked by bath application of glutamatergic agonists (D-glutamate, kainate or NMDA with or without D-serine; e.g., lamprey: Harris-Warrick and Cohen, 1985; Schotland and Grillner, 1993; salamander: Branchereau et al., 2000; rat: Pearlstein et al., 2005) or by brainstem stimulation (Dunbar et al., 2010). However, exogenous 5-HT was also found to increase rhythm frequency (e.g., rat: Cazalets et al., 1992). Such discrepancies may be dependent on the excitability state of the spinal circuits (Sharples and Whelan, 2017) or the recruitment of different 5-HT receptors (for review Perrier and Cotel, 2015, see also “Endogenous 5-HT” section). Exogenous 5-HT alone can evoke fictive locomotor activity in neonatal rats (Cazalets et al., 1992) or mice (e.g., Branchereau et al., 2000; Madriaga et al., 2004), and this is reminiscent of the results obtained in rabbits with 5-HT precursors (see “5-HT Precursors” section). Exogenous 5-HT increases the stability of left-right alternation and flexor/extensor alternations during fictive locomotion in the spinal cord of neonatal rats (Pearlstein et al., 2005) and mice (Dunbar et al., 2010).

At the cellular level, the effects of exogenous 5-HT on the locomotor circuitry are well documented. Many cellular mechanisms are involved in the effects of 5-HT. In lamprey, exogenous 5-HT: (i) decreases the slow afterhyperpolarization in motoneurons, ipsilaterally projecting excitatory interneurons and commissural interneurons and depresses synaptic transmission from premotor interneurons to motoneurons (El Manira et al., 1994; Wikström et al., 1995; Parker and Grillner, 1999; Hill et al., 2003; Biró et al., 2006; Wang et al., 2011, 2014; Pérez et al., 2013); (ii) depresses synaptic transmission from reticulospinal to spinal neurons through regulation of synaptic vesicle fusion (Blackmer et al., 2001; Schwartz et al., 2005, 2007; Photowala et al., 2006; Gerachshenko et al., 2009); and (iii) prolongs the depolarization plateau of NMDA-dependent oscillations of the membrane potential (Wallen et al., 1989). In zebrafish, the slow down of fictive locomotion evoked by exogenous 5-HT is linked to a stronger midcycle inhibitory drive and a slower build-up of the following excitatory drive in spinal neurons (Gabriel et al., 2009). In mouse, exogenous 5-HT increases the excitability and evokes bistable properties in V2a neurons (a class of locomotor glutamatergic interneurons, Zhong et al., 2010; Dietz et al., 2012; Husch et al., 2015). In mouse commissural neurons, exogenous 5-HT reduces calcium-activated potassium currents by decreasing N- and P/Q-type calcium currents (Díaz-Ríos et al., 2007; Abbinanti and Harris-Warrick, 2012; Abbinanti et al., 2012). In rat motoneurons, exogenous 5-HT facilitates the expression of NMDA-dependent oscillations of the membrane potential probably by regulating Mg^2+^ blockade at the NMDA ionophore (Maclean et al., 1998; MacLean and Schmidt, 2001). In lamprey reticulospinal neurons, exogenous 5-HT decreases the strength of synaptic responses evoked by stimulation of the trigeminal nerve and sensory relay cells (Antri et al., 2008).

### Endogenous 5-HT

5-HT is recaptured at the synapse by the 5-HT transporter (SERT) that can be blocked by SSRI, which increase the concentration of 5-HT at the synapse. Intriguingly, the effects of SSRI can be different from those of exogenous 5-HT. Whereas exogenous 5-HT increases rhythm stability, several studies reported that SSRI destabilizes fictive locomotion generated by the isolated spinal cord (lamprey: Christenson et al., 1989; mouse: Dunbar et al., 2010; zebrafish: Montgomery et al., 2018; salamander: Flaive et al., 2020). In some cases, in the same species, exogenous 5-HT decreases the frequency of fictive locomotor movements, whereas increasing the extracellular concentration of endogenous 5-HT increases the frequency (e.g., frog embryo: McLean and Sillar, 2004; salamander: Branchereau et al., 2000; Flaive et al., 2020). Whether this also occurs in other vertebrate classes remains to be explored.

The reasons why exogenous 5-HT and SSRI differentially affect fictive locomotion or the neurons of the locomotor circuitry are not fully resolved. From one study to another, the brainstem can be left attached to the spinal cord or removed from the preparation, possibly contributing to the variable outcomes of the experiments (Branchereau et al., 2000; Dunbar et al., 2010; Flaive et al., 2020). Another interesting possibility to consider is that different 5-HT receptors are recruited by exogenous or endogenous 5-HT (Dunbar et al., 2010; Cotel et al., 2013; for review Perrier and Cotel, 2015). The 5-HT system comprises 15 receptor subtypes, some of which have opposed effects on cell excitability. In spinal motor circuits, 5-HT_1_ receptors often decrease excitability, whereas 5-HT_2_ receptors often increase excitability (for review Perrier and Cotel, 2015). These receptors can be distributed at various distances from 5-HT release sites, making their activation dependent on the level of 5-HT released (Cotel et al., 2013; Perrier and Cotel, 2015). In the turtle spinal cord, short electrical stimulation (1–4 s train) of the descending 5-HT raphe fibers in the dorsolateral funiculus increase motoneuron excitability through 5-HT_2_ receptors, which activate a persistent inward current dependent on L-type calcium channels (Perrier and Delgado-Lezama, 2005). In contrast, prolonged electrical stimulation (3 min train) results in a spillover of 5-HT out of the synaptic site, activating 5-HT_1A_ receptors located at the axon initial segment, which depress motoneuron firing by inhibiting the sodium channels generating action potentials (Cotel et al., 2013). In line with this, prolonged activation of descending 5-HT fibers (1 min train) depresses fictive scratching in turtles, and this effect is blocked by a 5-HT_1A_ antagonist (Perrier et al., 2018). In rats, fictive locomotor activity evoked by dorsal root stimulation is deteriorated by 5-HT_1A_ receptor activation (Oueghlani et al., 2020). SSRI-evoked destabilization of fictive locomotion involves 5-HT_1A_ receptor activation in mouse (Dunbar et al., 2010) and salamanders (Flaive et al., 2020). Anatomical data in salamanders suggest that in addition to motoneurons, premotor glutamatergic interneurons are also a target of 5-HT release (Flaive et al., 2020). However, in mouse spinal cord slices, exogenous 5-HT induces a robust excitatory effect on the excitability of the V2a interneurons whereas citalopram has no effect (Husch et al., 2012). 5-HT release in slices may be different from that occurring in the intact spinal cord and this could contribute to this paradox (Dankoski and Wightman, 2013). Future studies are needed to determine the cellular basis of the difference between the effects of 5-HT and SSRI on fictive locomotor activity.

*In vivo*, SSRI have, overall, inhibitory effects on locomotor activity. In zebrafish larvae, bath-applied drugs pass the skin and access the neural system. Acute bath-application of SSRI decreases the distance traveled in a concentration-dependent manner in light/dark alternated swim tests (Bachour et al., 2020). Long term effects were also reported. After 3 weeks of exposure, fish stop swimming less often, and swimming velocity decreases (Nielsen et al., 2018). In humans, SSRI elicit excitatory effects on motor output during non-fatiguing exercise, but inhibitory effects emerge as fatigue progresses, supporting the idea that 5-HT plays a role in central fatigue (Kavanagh et al., 2019). Part of these effects likely involves 5-HT_1A_ receptors, which depress motoneuron excitability in humans (D’Amico et al., 2017). In humans with incomplete spinal cord injury, the use of SSRI was not found to improve walking performance, but rather to decrease stride length (Thompson and Hornby, 2013; Leech et al., 2014).

### Genetic Ablations

Because genetic tools are mostly available in rodents and zebrafish, the present section as well as the three following ones focus essentially on studies in these animal models. 5-HT neurons can be targeted for specific genetic deletions. 5-HT neurons express molecular markers such as the 5-HT transporter (*SERT*) gene (mouse: Seo et al., 2019), the *erythroblast transformation-specific domain (Pet1*) gene (a transcription factor expressed in 5-HT neurons, rat: Hendricks et al., 1999; zebrafish: Lillesaar et al., 2009; Montgomery et al., 2016) or the *Tryptophan hydroxylase 2 (Tph2)* gene, an enzyme involved in the synthesis of 5-HT (e.g., zebrafish: Yokogawa et al., 2012; mouse: Ikoma et al., 2018). When genetically ablating SERT, 5-HT is not recaptured at the synapse anymore, therefore increasing extracellular 5-HT. These animals were reported either to show normal locomotion in chambers equipped with photocell beams (Bengel et al., 1998) or hypolocomotion when placed in an observation cylinder (Kalueff et al., 2007).

When genetically ablating Pet1 in mice, 5-HT systems largely fail to develop (Hendricks et al., 2003). Unexpectedly, mice display normal locomotor activity in an open field arena (Hendricks et al., 2003; see also Zhao et al., 2006). The isolated spinal CPG generates normal fictive locomotor activity, although paradoxically exogenous 5-HT disrupts fictive locomotor activity in Pet1 knock-out mice, and this was linked to an increased sensitivity to 5-HT (Pearlstein et al., 2011). No major effect on locomotor behavior was found when ablating Tph2 neurons in zebrafish (Yokogawa et al., 2012). Altogether this suggests that the loss of 5-HT neurons can be compensated functionally. Whether the proportion of 5-HT receptors showing constitutive activity (i.e., spontaneously active intracellular signaling pathways in the absence of 5-HT, e.g., 5-HT2C receptors, see De Deurwaerdère et al., 2004; Navailles et al., 2006) increases in the absence of 5-HT in Pet1 knock-out mice remains to be determined (Pearlstein et al., 2011). This would be consistent with the observation that constitutively active 5-HT_2C_ receptors are more abundant after spinal injury (Murray et al., 2010), i.e., in conditions where the 5-HT innervation of the spinal cord is dramatically decreased, as it is the case in Pet1 knock-out mice (Pearlstein et al., 2011). Whether subtle effects on the locomotor pattern occur *in vivo* in Pet1 knock-out mice also remains to be determined.

### Manipulating Spinal 5-HT Neuron Activity

In the isolated spinal cord of zebrafish, optogenetic activation of Pet1-positive 5-HT neurons/fibers decreases the number of fictive locomotor bursts, an effect similar to that evoked by SSRI and exogenous 5-HT in the same preparation (Montgomery et al., 2018). It remains to be determined whether these effects involve only intraspinal 5-HT neurons and/or descending raphe 5-HT fibers that may still be able to release 5-HT in the isolated spinal cord (Montgomery et al., 2018).

### Recording and Manipulating 5-HT Neuron Activity in the Brainstem Caudal Group

Pioneering electrophysiological recordings of 5-HT neurons in the raphe obscurus and pallidus (which both mainly send descending projections to the spinal cord; Figure 1C) uncovered a positive correlation between locomotor speed and the spiking activity of 5-HT neurons (Jacobs and Fornal, 1993; Veasey et al., 1995), suggesting that an increased release of 5-HT in the spinal cord is associated with an increase in speed. 5-HT released in the spinal cord peaks after a few seconds during MLR-evoked locomotor bouts or shortly after, as shown by voltammetry recordings in the lumbar spinal cord in decerebrated cats (Noga et al., 2017). This suggests that 5-HT release modulates in the spinal cord the effects of the descending reticulospinal glutamatergic drive generated by the MLR. In rats, electrical or chemical stimulation of the parapyramidal region evokes fictive locomotion (Liu and Jordan, 2005). This region contains the para-raphe 5-HT neurons located in the mid-medulla oblongata, lateral to the nuclei raphe obscurus and raphe pallidus, and lateral to the pyramidal tract. The evoked fictive locomotor activity is blocked by 5-HT_2A_ or 5-HT_7_ receptor antagonists applied over the spinal cord (Liu and Jordan, 2005; for review Jordan et al., 2008). Stress-evoked hyperlocomotion is associated with increased activation of Tph2-positive 5-HT neurons in the medullary raphe nuclei as shown by the labeling of a cellular activation marker protein (the phosphorylated form of extracellular signal-regulated kinase, pERK; Ikoma et al., 2018). Optogenetic inhibition of Tph2-positive 5-HT neurons decreases stress-evoked hyperlocomotion, suggesting that modulation of locomotor behavior by stress is mediated by 5-HT neurons of the medullary raphe nucleus (Ikoma et al., 2018).

### Recording and Manipulating 5-HT Neuron Activity in the Brainstem Rostral Group

The relation between the activity of 5-HT neurons in the dorsal raphe nucleus and locomotor activity is complex and somewhat controversial (Cardozo Pinto et al., 2019). Short- and long-term effects were reported in mice. Acute chemogenetic activation of Pet1-positive neurons in the dorsal raphe nucleus reduces the distance traveled in an open field arena (Teissier et al., 2015). Acute optogenetic stimulation of Tph2-positive neurons decreases the distance traveled in the elevated plus-maze (Ohmura et al., 2014). Acute optogenetic stimulation of SERT-positive neurons rapidly stops locomotion in the open field arena (Correia et al., 2017). However, chronic stimulation (15 min per day for 3 weeks) increased the speed of spontaneous locomotion in an open field arena (Correia et al., 2017).

Recent studies indicate that 5-HT neurons in the dorsal raphe nucleus exert a context-dependent control in the regulation of behaviors involving locomotor activity in mice. Optogenetic stimulation of SERT-positive 5-HT neurons stops locomotion in an open field arena, but not in a linear track exposed to a bright light with water rewards at each end, and does not impair rotarod performance in mice (Correia et al., 2017). In another study, “fight-or-flight” responses were evoked in mice by modulating the level of threat in their environment (Seo et al., 2019). In low-threat environmental conditions such as the open field, the activity of SERT-positive 5-HT neurons decreases upon movement onset as shown by *in vivo* calcium imaging, and optogenetic activation of these 5-HT neurons decreases locomotor activity (Seo et al., 2019). In high-threat environmental conditions such as the tail suspension test, the activity of these 5-HT neurons increases upon escape movement onset, and optogenetic activation of these 5-HT neurons increases mobility (Seo et al., 2019). This behavioral response is reminiscent of the well-known effects of SSRI (Cryan et al., 2002). Altogether, this suggests that dorsal raphe 5-HT neurons can stop or facilitate movement in a context-dependent manner.

## Concluding Remarks

We provided an overview of the multiple layers of complexity that underlie the effects of 5-HT on locomotor activity. The locomotor circuitry is modulated by 5-HT at spinal and supraspinal levels. A diversity of 5-HT-dependent mechanisms at the cellular level was reported, such as reduction of a spike after afterhyperpolarization, depression of the synapse strength from interneurons to motoneurons, depression of the reticulospinal synapse, recruitment of persistent inward current in spinal neurons, or inhibition of sodium and calcium channels in spinal neurons.

Although not reviewed here, 5-HT also modulates sensory circuits both at spinal and brainstem levels, and depression of sensory transmission was reported in most, but not all cases (Antri et al., 2008; and references therein, for review Lopez-Garcia, 2006; Daghfous et al., 2016). Therefore, each 5-HT pharmacological agent likely has a complex profile of motor and sensory effects, based on the targeted receptors. The sensory targets of these molecules may also be important when aiming at reactivating the locomotor circuitry after spinal cord injury (Sławińska et al., 2014a; for review Sławińska et al., 2014b; Perrin and Noristani, 2019), given the importance of sensory feedback in CPG reactivation (for review Frigon, 2017).

The tools used to target the 5-HT system likely influence the outcome of the experiments. Tonic pharmacological stimulation with exogenous or endogenous 5-HT possibly recruits different 5-HT receptors, located close or far from 5-HT release sites (Perrier and Cotel, 2015). Short vs. prolonged activation of 5-HT fibers can end up in opposed effects possibly for a similar reason. Acute vs. chronic exposure to tonic pharmacological stimulation with SSRI produce different effects, and this is also the case when using acute vs. chronic phasic stimulation with optogenetics (Correia et al., 2017). Optogenetic or chemogenetic stimulation of 5-HT neurons evokes 5-HT release, but also possibly the release of co-transmitters, which are present in some 5-HT neurons (e.g., some SERT-positive 5-HT neurons in the dorsal raphe nucleus co-express the vesicular glutamatergic transporter 3 and co-release glutamate, Wang et al., 2019). 5-HT neurons also co-express numerous neuropeptides (Maxwell et al., 1996).

The maturation of intrinsic properties in neurons of the locomotor circuitry, of the level of innervation of the spinal cord, and 5-HT receptor expression levels in different parts of the locomotor network also contribute to the variability of 5-HT effects during development (for review Jean-Xavier et al., 2018; Hachoumi and Sillar, 2020). In the tadpole, 5-HT has different effects at early and late larval stages (for review Sillar et al., 2014). In mice, adult V2a interneurons are more sensitive to 5-HT than neonatal ones, and the maturation of intrinsic properties in V2a neurons leads to the emergence of 5-HT-induced bistable properties in adults (Husch et al., 2015).

Future studies should determine whether spinal 5-HT neurons indeed modulate the locomotor circuitry (Montgomery et al., 2018) and/or play a paracrine function by releasing 5-HT in the extraspinal space (Schotland et al., 1996; Chiba, 2007). It should also be examined whether some 5-HT neurons of the brain caudal group (Figure 1B) are part of the brainstem locomotor circuitry. Interestingly, the lateral medullary formation (LPGi), which contains many 5-HT neurons (Hornung, 2010), also contains glutamatergic reticulospinal neurons that play a key role in relaying the locomotor drive from the MLR to the spinal cord in mice (Capelli et al., 2017). Whether these reticulospinal neurons are 5-HT-positive is not known. Some Pet1-positive 5-HT neurons were recently described in the pedunculopontine nucleus, the ventral part of the MLR, but their role in locomotor control remains to be determined (Ge et al., 2019). The role of the 5-HT innervation of the cerebellum in locomotor control is unknown but interestingly: (i) all levels of the cerebellum receive 5-HT fibers; (ii) 5-HT modulates the electrophysiological activity of many cell types in the cerebellum; (iii) the level of cerebellar 5-HT increases during motor activity; and (iv) 5-HT cell metabolism abnormalities were detected in cerebellar ataxia (for review Trouillas, 1993; Schweighofer et al., 2004).

Context-dependent modulation of locomotor output by the same 5-HT nucleus suggests that different 5-HT cell subtypes in the dorsal raphe nucleus are recruited in a behavior-dependent manner (Correia et al., 2017; Seo et al., 2019). Such different cell subtypes likely exist in this nucleus, as recently uncovered by single-cell transcriptomics, anatomical and electrophysiological properties (Ren et al., 2018; Cardozo Pinto et al., 2019; Huang et al., 2019; Okaty et al., 2020). These likely project to different brain regions involved in modulating the locomotor circuitry. Among the numerous possible targets of these 5-HT neurons (Hornung, 2010), the basal ganglia, cortex, and dopaminergic neurons are largely innervated by 5-HT fibers and express a variety of 5-HT receptors (for review De Deurwaerdère and Di Giovanni, 2017; Vitrac and Benoit-Marand, 2017). Future studies should identify whether different 5-HT cell populations have distinct projections patterns to these structures and determine whether this underlies context-dependent modulation of locomotor activity.

## Author Contributions

AF, MF, CZ, and DR wrote the manuscript. All authors contributed to the article and approved the submitted version.

## Conflict of Interest

The authors declare that the research was conducted in the absence of any commercial or financial relationships that could be construed as a potential conflict of interest.
